# Evaluation of the Effect of *Astragalus membranaceus* Saponins Administration on Knee Function and Cartilage Biomarkers in Healthy Subjects with Knee Discomfort

**DOI:** 10.3390/nu18121842

**Published:** 2026-06-07

**Authors:** Shu Ru Zhuang, Pui-Ying Leong, Hsin-Pei Chiang, You-Cheng Shen

**Affiliations:** 1 Department of Nutrition, Chung Shan Medical University, Taichung 402, Taiwan; cristine0131@gmail.com; 2 Institute of Medicine, Chung Shan Medical University, Taichung 402, Taiwan; pyleong@csmu.edu.tw; 3 Division of Allergy, Immunology and Rheumatology, Department of Internal Medicine, Chung Shan Medical University Hospital, Taichung 402, Taiwan; 4 PhD Program of Business, Feng Chia University, Taichung 402, Taiwan; 5 Department of Health Industry Technology Management, Chung Shan Medical University, Taichung 402, Taiwan; workingpei7@gmail.com; 6 Department of Nutrition, Chung Shan Medical University Hospital, Taichung 402, Taiwan

**Keywords:** *Astragalus membranaceus* saponins, knee discomfort, joint function, KOOS, range of motion, biomarkers, randomized controlled trial

## Abstract

Objective: This study aimed to evaluate the effects of 12 weeks of *Astragalus membranaceus* saponins (AMS) supplementation on functional performance, knee joint mobility, self-reported outcomes, and biomarkers of inflammation and cartilage in healthy subjects with knee discomfort. Methods: A randomized, double-blind, placebo-controlled trial was conducted in healthy subjects aged 20–70 years with knee discomfort but without clinically diagnosed knee osteoarthritis. Participants were assigned to receive one capsule of AMS or a placebo once daily for 12 weeks. The pre-specified primary endpoints were the SLSD step count and knee ROM; KOOS total score was a key secondary endpoint; serum biomarkers were exploratory. The results included functional performance assessed by the Single Leg Step Down (SLSD) test, knee range of motion (ROM), and self-reported outcomes using the Knee injury and Osteoarthritis Outcome Score (KOOS). Knee ROM was measured with a goniometer and recorded as both active ROM and passive ROM for knee flexion and extension. Serum biomarkers of inflammation (IL-8, IL-1β, MIP-1α), cartilage degradation (CTX-II, COMP, MMP-13, COL2A1), and cartilage synthesis (PIINP) were evaluated at baseline and Week 12. Results: Within the AMS group, SLSD step count increased significantly by 16.83% (Δ = +12.78 steps; *p* < 0.05) and recovery time decreased significantly by 19.12% (Δ = −108.91 s; *p* < 0.05) compared with baseline, whereas the placebo group showed smaller, non-significant changes (+4.48 steps and −56.48 s, respectively); however, neither between-group difference in change scores reached statistical significance. Significant improvements in active and passive knee ROM were observed in both flexion and extension (all *p* < 0.05) within the AMS group, whereas the placebo group showed no significant changes. KOOSs improved significantly in all domains within the AMS group, with the largest gains observed in sport/recreation (+22.23%) and quality of life (+18.38%). In the exploratory biomarker analysis, several inflammation and cartilage-related biomarkers changed after AMS supplementation showed within-group reductions (IL-8, COMP, MMP-13) and PIINP increased. Conclusions: 12 weeks of AMS supplementation was associated with improvements in selected functional, mobility, and outcomes in generally healthy adults with self-reported knee discomfort. AMS was also associated with changes in selected circulating biomarkers related to inflammation and cartilage metabolism. These findings should be interpreted as a preliminary, safe, complementary strategy to support joint health in healthy subjects with knee discomfort.

## 1. Introduction

Maintaining knee joint mobility is important for both physical performance and everyday function [1,2]. Even in healthy and physically active adults, however, repetitive mechanical loading may lead to activity-related knee discomfort, which often presents as transient pain, stiffness, and mild reductions in knee range of motion (ROM) despite the absence of diagnosed joint disease [3,4]. Standardized loading tasks, such as the Single Leg Step Down (SLSD) test, are widely used to evaluate joint-related functional stress and movement control. In combination with range-of-motion (ROM) measurements, they may also help detect early functional limitations relevant to both daily activities and sports participation [5,6,7]. Because even subtle declines in joint mobility and movement quality may adversely affect gait mechanics and physical performance, nutritional strategies that help preserve joint comfort and function are of growing interest.

In recent years, nutritional and nutraceutical interventions have been increasingly investigated as accessible approaches for supporting joint health across a spectrum ranging from mild discomfort to symptomatic joint disorders [8,9,10]. Glucosamine has long attracted attention because it serves as a precursor for glycosaminoglycans and contributes to the synthesis of hyaluronic acid, both of which are essential for the structural integrity and biomechanical function of the extracellular matrix of cartilage [11,12,13]. Similarly, collagen-based ingredients, including collagen peptides and non-denatured type II collagen, have shown the potential to improve joint-related symptoms and physical function in clinical studies [14,15,16,17]. More recently, botanical bioactives have emerged as promising candidates for joint health support. Among these, *Astragalus membranaceus*, a traditional medicinal herb, contains cycloartane-type saponins with reported anti-inflammatory, antioxidant, and tissue-protective activities [18,19]. Preclinical findings suggest that *Astragalus membranaceus* saponins (AMS) may support cartilage homeostasis through multiple mechanisms, including enhancement of glucosamine uptake, increased hyaluronic acid secretion, promotion of proline uptake, restoration of collagen II expression under inflammatory conditions, and suppression of matrix metalloproteinases involved in cartilage degradation [20]. Collectively, these observations support the hypothesis that AMS may exert joint-protective effects by promoting extracellular matrix synthesis while attenuating inflammation-related matrix breakdown.

Despite these promising mechanistic findings, the clinical evidence remains limited and heterogeneous. In particular, relatively few randomized controlled trials have examined whether nutritional supplementation can improve knee discomfort in healthy subjects who are otherwise healthy and physically active, rather than in patients with established osteoarthritis [8,21]. In addition, self-reported symptoms, functional performance, and objective mobility outcomes are not always evaluated together, making it difficult to determine whether perceived improvements are accompanied by meaningful functional benefits [8,22]. Biomarker findings are also inconsistent across studies, and it remains unclear whether symptomatic or functional improvements are paralleled by favorable changes in systemic inflammation and markers of cartilage degradation or synthesis [21,22,23].

Accordingly, the present study investigated the effects of 12 weeks of AMS supplementation in adults with knee discomfort using a randomized, double-blind, placebo-controlled design. Functional performance was assessed using the SLSD test, knee mobility was evaluated by goniometric measurement of active and passive ROM, and self-reported symptoms and function were assessed using the Knee injury and Osteoarthritis Outcome Score (KOOS). In addition, serum biomarkers related to inflammation, cartilage degradation, and cartilage synthesis were analyzed to determine whether AMS supplementation was associated with coordinated changes in subjective outcomes, physical function, and biological indicators. The study hypothesized that AMS supplementation would improve knee-related symptoms and functional performance and would be accompanied by favorable changes in biomarkers related to cartilage integrity and inflammation.

## 2. Materials and Methods

### 2.1. Study Design

This study was a 12-week, randomized, double-blind, placebo-controlled, parallel-group clinical trial designed to evaluate the effects of AMS supplementation on knee discomfort, functional outcomes, and cartilage-related biomarkers. Randomization was performed using a computer-generated sequence, and allocation was concealed with sealed opaque envelopes. Both participants and study personnel responsible for outcome assessments and data analysis were blinded to group assignments.

### 2.2. Sample

AMS is a botanical saponin-rich extract of *Astragalus membranaceus* (20:1 aqueous extract) roots blended with maltodextrin as an excipient; its production is compliant with current Good Manufacturing Practice. The final blend is light brown to brown, yellow powder, and is standardized to contain ≥3% saponins. TLC/HPTLC fingerprinting was performed using astragaloside IV as a reference marker. Samples were applied to silica gel 60 HPTLC plates and developed with chloroform–methanol–distilled water (30:8:1, *v*/*v*/*v*), followed by derivatization with 10% ethanolic sulfuric acid and heating at 105 °C. The AMS sample showed a band co-migrating with astragaloside IV, supporting the presence of characteristic Astragalus saponins. The fingerprint is shown in Supplementary Figure S2. The AMS used in this study was provided by NuLiv Science USA, Inc. (Brea, CA, USA). The AMS capsules consisted of 200 mg of the proprietary extract, and the placebo capsules contained maltodextrin.

### 2.3. Participants

**Figure 1:**
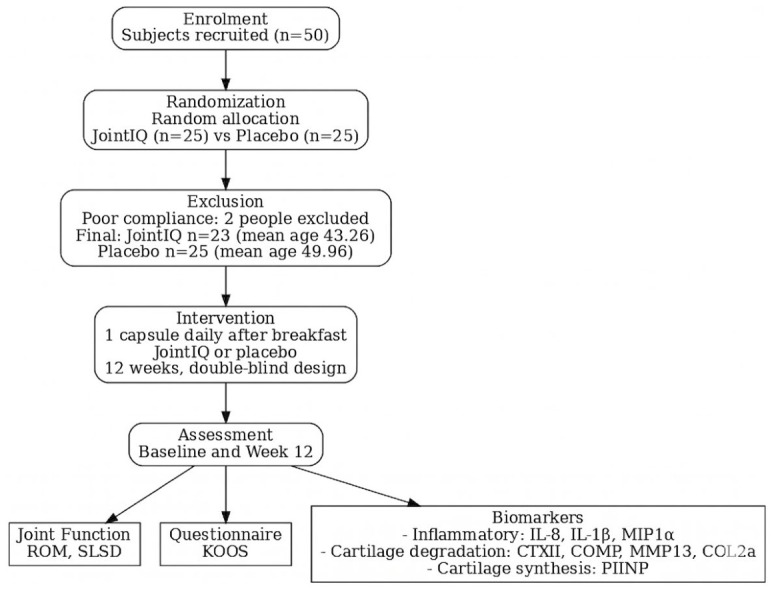
Recruitment process flow chart.

A total of 50 healthy subjects with self-reported knee discomfort were recruited. After excluding participants with poor compliance, 23 subjects remained in the AMS group (mean age: 43.26 years) and 25 in the placebo group (mean age: 49.96 years) (Figure 1). Eligible participants were men or women aged 18–75 years with self-reported knee discomfort who experienced pain of at least 5 points on the visual analog scale (VAS) during the Single Leg Step Down (SLSD) test when performing 30 to 150 steps (Supplementary Table S1). Exclusion criteria included recent knee surgery (within six months), intra-articular corticosteroid or hyaluronic acid injections in the three months, active inflammatory joint diseases, systemic infection, or use of investigational products in the three months, clinically diagnosed knee osteoarthritis, body mass index ≥ 35 kg/m^2^, joint effusion, or evident structural deformity on physical examination. The study population was not defined by radiographic, ultrasound, or MRI evidence of joint pathology. The study protocol was approved by the Institutional Review Board of Chung Shan Medical University Hospital (Protocol No. CS2-24092) and registered at ClinicalTrials.gov (NCT07382622). Written informed consent was obtained from all participants prior to enrollment. All participants provided written informed consent, and the study was conducted in accordance with the Declaration of Helsinki.

### 2.4. Interventions

Participants were instructed to consume one capsule of AMS or a placebo once daily after breakfast for 12 weeks. The AMS capsules contained the proprietary active formulation, whereas the placebo capsules contained inert excipients but were identical in appearance, taste, and packaging. Adherence was monitored through capsule counts at follow-up visits. Participants were advised to maintain their usual diet and physical activity and to avoid new exercise programs or joint-related supplements during the trial (Supplementary Table S1).

### 2.5. Outcome Measurements

Endpoint hierarchy. The primary endpoints were the SLSD step count and knee ROM (active and passive, flexion and extension). The key secondary endpoint was the KOOS total score. All serum inflammation and cartilage-related biomarkers (IL-8, IL-1β, MIP-1α, CTX-II, COMP, MMP-13, COL2A1, PIINP) were pre-specified as exploratory and were analyzed.

#### 2.5.1. Functional Assessments

The Single Leg Step Down (SLSD) test is a unilateral functional performance assessment designed to evaluate knee joint discomfort during weight-bearing activity. Testing was performed on the limb typically associated with greater pain following physical activity (target knee). Participants stood on a platform adjusted in height according to individual shank length (shank length ÷ 2.3 cm) and performed continuous, controlled step-downs at a pace of approximately 3 s intervals, to a maximum of 200 repetitions. During each repetition, the descending limb’s heel briefly contacted the floor before the participant returned to full knee extension on the platform. Throughout the test, participants continuously rated knee discomfort using a visual analog scale (VAS; 0 = “no pain,” 10 = “worst pain possible”). The test was terminated when participants reported a VAS pain score of 5. The total number of repetitions completed was recorded as the primary outcome. Additionally, the time from test completion to full recovery from knee joint pain was documented as a secondary outcome. SLSD measurements were performed by trained assessors blinded to treatment allocation. The same assessor measured the same participant at baseline and Week 12.

#### 2.5.2. Knee Range of Motion (ROM)

Knee flexion and extension were measured using a goniometer under both active (voluntary effort) and passive (assisted by the examiner) conditions. Angles were recorded in degrees at baseline and Week 12. ROM measurements were performed by trained assessors blinded to treatment allocation using standardized anatomical landmarks and goniometer placement. The same assessor measured the same participant at baseline and Week 12. Measurements were repeated according to the standardized protocol to reduce measurement variability.

#### 2.5.3. Self-Reported Outcomes

The Knee injury and Osteoarthritis Outcome Score (KOOS) questionnaire was administered to assess symptoms, pain, activities of daily living, sport and recreation, and knee-related quality of life. Higher scores reflect better joint function and less discomfort.

#### 2.5.4. Biomarker Analysis

Fasting blood serum samples were collected at baseline and Week 12. Inflammatory cytokines measured included interleukin-8 (IL-8), interleukin-1β (IL-1β), and macrophage inflammatory protein-1α (MIP-1α). Cartilage degradation markers included the C-terminal telopeptide of type II collagen (CTX-II), cartilage oligomeric matrix protein (COMP), matrix metalloproteinase-13 (MMP-13), and the type II collagen alpha-1 chain (COL2A1). The cartilage synthesis marker procollagen type II N-terminal propeptide (PIINP) was also quantified. All biomarkers were analyzed using commercially available enzyme-linked immunosorbent assay (ELISA) kits according to manufacturer instructions, with duplicate measurements performed to ensure reliability. Intra-assay coefficients of variation (CV) were <8% and inter-assay CVs were <12% for all assays. The number of samples with values within 2 × LoD is reported in Supplementary Table S2. Boxplots with individual data points for all biomarkers are provided as Supplementary Figure S1.

### 2.6. Statistical Analysis

Data were expressed as mean ± standard error (SE). The Shapiro–Wilk test was applied to assess normality. The principal analysis was a between-group comparison of change scores (Δ Week 12 − Week 0), evaluated using an independent *t*-test, with mean change ± SE. Within-group changes (paired-sample *t*-tests or Wilcoxon signed-rank tests, depending on normality) are reported as supportive information. Because the eight serum biomarkers were pre-specified as exploratory, two-sided *p*-values across this panel were adjusted using the Benjamini–Hochberg false-discovery-rate procedure, and corrected q-values are reported in Supplementary Table S3. Statistical analyses were conducted using SPSS version 20.0 (SPSS Inc., Chicago, IL, USA).

## 3. Results

### 3.1. Functional Performance of the Knee

#### 3.1.1. The Single Leg Step Down (SLSD) Test

SLSD is an appropriate test to induce joint discomfort in subjects with activity-related joint discomfort of the knee. Activity-related joint discomfort is a common problem in daily life of physically active people, as this may limit their mobility and flexibility as well as physical performance. After 12 weeks of supplementation, the AMS group showed significant improvements within-group in both step count and pain recovery time compared with baseline, while no statistically significant changes were observed in the placebo group (Table 1).

The step count increased significantly by 19.66% in the AMS group compared with baseline (week 0: 65.00 ± 5.06; week 12: 77.78 ± 7.44, *p* < 0.05). In contrast, the placebo group showed a nonsignificant increase of 6.41% (week 0: 69.92 ± 7.55; week 12: 74.40 ± 6.71, *p* = 0.346). Moreover, the time required for complete recovery from knee joint pain significantly decreased by 33.60% in the AMS group compared with baseline (week 0: 324.14 ± 37.70 s; week 12: 215.23 ± 28.91 s, *p* < 0.001), whereas the placebo group showed a nonsignificant 22.36% reduction (week 0: 252.56 ± 38.40 s; week 12: 196.08 ± 19.25 s, *p* = 0.108).

However, neither between-group difference reached statistical significance: step count increased by 12.29% in the AMS group compared with the placebo group (Δ = +8.30 steps; *p* = 0.221), and completion time decreased by 18.33% (Δ = −52.43 s; *p* = 0.220).

Collectively, the AMS group showed greater improvements than the placebo group in both SLSD step count and recovery time. However, the between-group differences in change scores did not reach statistical significance in this trial. These findings suggest that AMS supplementation may have potential as a joint support nutraceutical for activity-related knee discomfort in both recreationally active and athletic populations. These results should be regarded as exploratory.

#### 3.1.2. Knee Range of Motion

The range of motion (ROM) measurement is a widely used method for the assessment of the flexibility of different joint types. The ROM measurement was performed under active and passive conditions. The knee ROM of a joint is typically measured by the number of degrees from the starting position of a segment to its position at the end of its full range of movement (Table 2).

#### 3.1.3. Active and Passive Knee Flexion ROM

After 12 weeks of supplementation, the AMS group showed significant within-group improvements in active knee flexion by 5.86% compared with baseline (week 0: 103.13 ± 3.72°; week 12: 109.17 ± 2.79°, *p* < 0.05) and in passive knee flexion by 3.82% (week 0: 110.48 ± 3.34°; week 12: 114.70 ± 2.61°, *p* < 0.05), as showed in Table 2. However, there was no significant difference in the placebo group compared with the baseline measurement. Between-group comparisons for knee flexion showed directionally positive but non-significant differences (Supplementary Table S3).

#### 3.1.4. Active and Passive Knee Extension ROM

AMS supplementation also led to significant within-group improvements in knee extension, as shown in Table 2. Active extension ROM increased by 13.25% compared with baseline (Week 0: 69.29 ± 2.88°; Week 12: 78.47 ± 1.31°, *p* < 0.05). Passive extension improved by 12.95% (Week 0: 73.59 ± 2.44°; Week 12: 83.12 ± 1.42°, *p* < 0.001). In the placebo group, changes in extension ROM were not statistically significant for either active (71.22 ± 1.63° to 74.70 ± 1.49°, *p* = 0.083) or passive extension (75.96 ± 1.73° to 79.00 ± 1.56°, *p* = 0.112).

In the between-group analysis, active extension also improved by 8.90% with a between-group difference approaching significance (Δ = +6.26°; *p* = 0.051). Passive extension improved significantly by 8.65% in the AMS group compared with the placebo group (Δ = +6.49°; *p* = 0.026). Although the statistical significance became attenuated after multiple-comparison correction, the magnitude of change exceeded the approximate 5° threshold previously suggested as clinically meaningful for joint mobility outcomes.

Taken together, the strongest functional signal observed in this trial was the improvement in passive knee extension ROM, which showed the largest functional effect and exceeded the established MCID of approximately 5°. Although the other ROM endpoints did not reach statistical significance, their changes also exceeded 5°, suggesting potentially clinically meaningful improvements in knee mobility and function among healthy subjects engaged in regular physical activity.

#### 3.1.5. Knee Injury and Osteoarthritis Outcome Score (KOOS)

The KOOS survey is a validated instrument consisting of 42 questions that are classified into five subscales, such as symptoms, pain, function in daily living, function in sport and recreation, and knee-related quality of life (Table 3). It measures the subjects’ opinions about their knees and their ability to perform daily activities during the past week. The score is a percentage score from 0 to 100, with 0 representing extreme problems and 100 representing no problems.

Compared to baseline measurement, participants in the AMS group exhibited significant within-group improvements across all KOOS parameters measured. Specifically, symptom score improved by 13.46%, pain scores improved by 12.32%, function in daily living increased by 8.38%, function in sport and recreation increased by 22.23%, and knee-related quality of life by 18.38%. The total KOOS also significantly increased by 14.32%.

In contrast, the placebo group did not exhibit significant changes in any of the KOOS subscales during the study period. Although some increases were observed (e.g., function in sport and recreation from 61.19 ± 3.62 to 66.43 ± 3.09), these were not statistically significant (*p* > 0.05).

In the between-group analysis of change scores, KOOS Quality of Life improved significantly by 21.86% in the AMS group compared with the placebo group (Δ = +11.70 points; *p* = 0.003), exceeding the published MCID range of 7–10 points and retaining significance after FDR correction (q = 0.038). KOOS Total score also improved significantly by 10.39% (Δ = +6.59 points; *p* = 0.010), as did KOOS Pain by 9.26% (Δ = +6.51 points; *p* = 0.020) (Supplementary Table S3).

These results further underscore the efficacy of AMS supplementation in reducing joint discomfort and enhancing functional outcomes.

### 3.2. Inflammatory Biomarkers

Following 12 weeks of AMS supplementation, IL-8 levels decreased significantly by 25.16% compared with baseline (week 0: 3.10 ± 0.53 pg/mL; week 12: 2.32 ± 0.36 pg/mL, *p* < 0.05). In contrast, the placebo group showed a non-significant decrease of 7.52% (*p* = 0.110). IL-1β levels in the AMS group declined by 17.15% compared with baseline (week 0: 878.62 ± 108.25 pg/mL; week 12: 727.91 ± 125.96 pg/mL; *p* = 0.344), whereas the placebo group showed a 4.27% increase (*p* = 0.791). MIP-1α levels showed a modest decrease of 3.47% in the AMS group (*p* = 0.822) and a 2.79% increase in the placebo group (*p* > 0.05), although these changes did not reach statistical significance (Table 4). In the between-group analysis of change scores, IL-8 levels in the AMS group decreased significantly by 19.86% compared with the placebo group (between-group Δ = −0.57 pg/mL; *p* = 0.049). In contrast, IL-1β and MIP-1α showed no significant between-group differences. Although the IL-8 signal did not survive multiple-comparison correction, it remains biologically meaningful: IL-8 plays a central role in chondrocyte inflammation.

These exploratory results provide a preliminary signal that AMS may influence IL-8-related chemokine pathways, which is a key pro-inflammatory cytokine involved in cartilage degradation and joint inflammation.

### 3.3. Cartilage Biomarkers

After 12 weeks of AMS supplementation, several cartilage-related biomarkers showed significant within-group changes compared with baseline, as shown in Table 5. CTX-II levels decreased by 50.00% compared with baseline (week 0: 2.19 ± 0.55 ng/mL; week 12: 1.10 ± 0.23 ng/mL, *p* < 0.05), COMP levels significantly declined by 47.81% (week 0: 4.56 ± 0.76 ng/mL; week 12: 2.38 ± 0.32 ng/mL, *p* < 0.05). MMP-13 levels were also significantly reduced by 25.23% (week 0: 2708.85 ± 299.98 pg/mL; week 12: 2025.44 ± 234.90 pg/mL, *p* < 0.05). Similarly, COL2A1 levels decreased by 23.24% (week 0: 7.66 ± 1.98 ng/mL; week 12: 5.88 ± 1.41 ng/mL). In contrast, the placebo group showed no significant changes in any of these biomarkers.

Regarding cartilage synthesis related biomarkers, PIINP levels in the AMS group increased significantly by 35.45% compared with baseline (week 0: 2.20 ± 0.30 ng/mL; week 12: 2.98 ± 0.33 ng/mL, *p* < 0.05), whereas the placebo group showed a non-significant increase of 11.67% (week 0: 1.80 ± 0.26 to week 12: 2.01 ± 0.26 ng/mL; *p* = 0.054).

In between-group comparisons, COMP levels in the AMS group decreased significantly by 49.41% compared with the placebo group (between-group Δ = −2.02 ng/mL; *p* = 0.049), representing the only marker with a statistically significant between-group difference. PIINP also demonstrated a directionally favorable between-group difference of 28.50% approaching significance (Δ = +0.57 ng/mL; *p* = 0.059). Although the statistical significance did not remain after multiple-comparison correction, these biomarkers may still reflect biologically relevant changes. COMP is a widely used marker of cartilage matrix turnover, whereas PIINP reflects type II collagen synthesis.

These findings suggest that AMS supplementation may promote cartilage health by both suppressing cartilage degradation and enhancing cartilage synthesis activity. Additionally, the increase in PIINP is consistent with a shift in matrix turnover markers, although structural confirmation by imaging remains necessary.

## 4. Discussion

This 12-week randomized, double-blind, placebo-controlled trial provides preliminary evidence that AMS supplementation may support selected aspects of knee function and joint-related quality of life in healthy adults with self-reported knee discomfort but without clinically diagnosed osteoarthritis. In the principal between-group analyses, AMS produced statistically significant and clinically meaningful improvements in passive knee extension ROM and the KOOS quality-of-life domain. Although the pre-specified primary endpoint, SLSD step count, did not show a significant between-group difference, the AMS group demonstrated within-group improvements in SLSD performance, recovery time, knee ROM, and KOOS domains. Several additional secondary outcomes, including KOOS pain, KOOS total score, and active knee extension, showed favorable effect sizes but should be interpreted cautiously because statistical significance was attenuated after correction for multiple comparisons. Exploratory biomarker analyses showed within-group reductions in IL-8 and cartilage degradation-related markers, including COMP and MMP-13, together with an increase in PIINP. However, these biomarker findings did not remain significant after FDR correction and should therefore be regarded as preliminary, hypothesis-generating signals rather than confirmatory evidence of anti-inflammatory or cartilage-modifying effects. Taken together, these findings suggest that AMS may represent a safe and promising phytochemical-based complementary strategy for supporting knee mobility, perceived joint-related quality of life, and cartilage metabolism.

Our findings are consistent with previous reports that demonstrate that dietary supplement interventions can improve physical function and quality of life in individuals with knee osteoarthritis or knee discomfort [8,24]. Similar to prior studies of collagen peptides, glycosaminoglycans, and other joint-support ingredients, the present improvements in SLSD performance, ROM, and KOOSs suggest that bioactive nutritional compounds may enhance joint stability, reduce functional limitation, and improve symptom perception [8]. Notably, the largest KOOS gains in the current study were observed in sport and recreation (+22.23%) and quality of life (+18.38%), suggesting that AMS may be particularly relevant for activities that impose greater mechanical demand on the knee joint. This pattern is clinically meaningful because functional deficits in otherwise healthy but symptomatic adults are often more apparent during demanding movements than during routine daily tasks. In addition, the significant reduction in IL-8 is consistent with evidence that nutraceutical interventions may attenuate low-grade inflammation by modulating chemokine-related pathways involved in neutrophil recruitment and cartilage catabolism [8,24].

The within-group reductions in COMP and MMP-13, together with the within-group increase in PIINP, are consistent with the previously reported in vitro mechanistic profile [20]. However, because none of these biomarkers remained statistically significant after multiple-comparison correction in the principal between-group analysis, the present clinical data is insufficient to conclude that AMS restores cartilage matrix homeostasis. The relatively healthy study population without clinically diagnosed osteoarthritis may also have limited the ability to detect robust between-group biomarker differences over the 12-week intervention period. Therefore, the observed biomarker pattern should be interpreted as preliminary mechanistic support for the concept that AMS may influence the balance between cartilage breakdown and matrix repair and should be validated in adequately powered trials.

The fact that these biomarker changes were accompanied by improved ROM and faster post-exertional recovery strengthens the clinical relevance of the observed biochemical effects [8]. Another noteworthy aspect of the present study is that AMS is a plant-derived bioactive compound, which distinguishes it from more widely studied joint-support ingredients such as animal-derived collagen peptides and glucosamine. In contrast, AMS is a botanical saponin-rich extract from *Astragalus membranaceus*, suggesting a different biological rationale for joint support. Rather than acting mainly as a structural precursor, AMS may exert its effects through modulation of inflammation-related signaling, suppression of cartilage catabolism, and support of extracellular matrix homeostasis [20]. This difference in origin and putative mechanism supports the scientific value of investigating AMS as a distinct nutritional strategy for joint health, particularly in the context of increasing interest in plant-based and complementary interventions.

The mechanistic basis for the observed benefits of AMS can be interpreted in light of previous in vitro evidence. Huang et al. [20] reported that AMS significantly enhanced glucosamine uptake in primary human chondrocytes, thereby increasing the intracellular availability of a key precursor for glycosaminoglycan and proteoglycan biosynthesis. Because glucosamine is a fundamental building block of glycosaminoglycans in the extracellular cartilage matrix, this mechanism may contribute to improved matrix maintenance [11]. AMS was also shown to increase hyaluronic acid secretion in chondrocytes, a finding of particular interest given the central role of hyaluronic acid in synovial fluid lubrication and maintenance of the pericellular matrix surrounding chondrocytes [12,13]. Furthermore, AMS promoted proline uptake, suggesting an improved capacity for collagen synthesis, since proline is a major amino acid component of collagen, the principal structural macromolecule of the extracellular cartilage matrix [20]. In an IL-1β-induced osteoarthritis model, AMS restored collagen II expression and suppressed the expression and proteolytic activities of MMP-1, MMP-2, MMP-9, and MMP-13, all of which are involved in cartilage matrix degradation [20]. These preclinical observations provide a coherent framework for the present clinical findings. Specifically, reductions in serum CTX-II, COMP, MMP-13, and COL2A1 may reflect suppression of cartilage-degradation pathways, whereas the increase in PIINP may indicate enhanced collagen synthesis or matrix remodeling [25,26]. The concurrent reduction in IL-8 is also consistent with the broader anti-inflammatory properties of Astragalus saponins, which have been reported to modulate inflammatory signaling and protect chondrocytes against cytokine-induced damage [27,28]. Taken together, these findings support a dual-action mechanism of AMS, characterized by the promotion of extracellular matrix biosynthesis (e.g., collagen II and hyaluronic acid) and the suppression of inflammation-induced cartilage catabolism (e.g., MMP activity), thereby contributing to the observed improvements in function, symptoms, and biomarker profiles.

This study makes several noteworthy contributions to the field of joint health research [8,29]. First, it applied a comprehensive outcome framework by integrating functional testing, self-reported outcomes, and biomarker analyses within the same randomized controlled trial [8,30]. This multidimensional approach strengthens the link between subjective improvement and objective physiological change, a relationship that is often insufficiently addressed in previous nutraceutical studies [31,32]. Second, the consistent pattern of improvement across clinical and molecular endpoints suggests that AMS may exert dual effects, namely suppression of inflammation-driven cartilage catabolism together with enhancement of anabolic processes related to collagen synthesis [8,30]. Such findings extend the current understanding of nutraceutical efficacy by suggesting the potential for joint-support benefits beyond symptom relief alone [23,31]. In addition, the present biomarker profile highlights IL-8, COMP, and PIINP as potentially useful indicators for monitoring short-term response to joint-targeted supplementation, supporting their possible value as surrogate endpoints in future interventional studies [29,30].

Despite these strengths, several limitations should be acknowledged. The relatively small sample size may have limited the statistical power to detect subtle between-group differences, particularly for IL-1β, MIP-1α, and certain KOOS domains. The 12-week duration was sufficient to identify short-term functional and biochemical changes, but it may not be long enough to determine whether AMS can influence long-term cartilage structure or osteoarthritis progression. In addition, imaging modalities such as MRI or ultrasound were not included and therefore direct structural evidence of cartilage preservation was not available. The study population also consisted of generally healthy adults with knee discomfort, and the findings may not be generalizable to patients with advanced osteoarthritis or substantial comorbidities. Therefore, future studies should include larger cohorts, longer intervention periods, imaging-based structural endpoints, and dose–response designs to better define the therapeutic and preventive potential of AMS. Comparative trials against other nutraceutical or pharmacological interventions may also help clarify its relative clinical value in joint health management.

## 5. Conclusions

In this 12-week randomized, double-blind, placebo-controlled trial, AMS supplementation produced statistically significant between-group improvements in two clinically meaningful endpoints, including passive knee extension ROM and KOOS quality of life. Several additional functional and self-reported outcomes showed moderate positive effects but did not reach statistical significance in this trial. Exploratory biomarker analyses identified within-group reductions in cartilage degradation marker COMP and an increase in PIINP. These findings indicate that AMS may represent a safe and promising phytocompound-based complementary strategy for supporting joint health in healthy subjects with knee discomfort.

## Figures and Tables

**Table 1:** Single-leg step-down test (SLSD).

		AMS	Placebo
SLSD (steps)	Week 0	65.00 ± 5.06	69.92 ± 7.55
	Week 12	77.78 ± 7.44 *	74.40 ± 6.71
Recovery time (s)	Week 0	324.14 ± 37.70	252.56 ± 38.40
	Week 12	215.23 ± 28.91 **	196.08 ± 19.25

Data are expressed as mean ± SE. Asterisks indicate within-group changes from Week 0 to Week 12 based on paired tests after BH-FDR correction: * *p* < 0.05, ** *p* < 0.01. Between-group comparisons were performed on change scores (Δ Week 12 − Week 0) and are reported in Supplementary Table S3.

**Table 2:** Knee range of motion (knee ROM).

	Time	AMS	Placebo
Active Knee Flexion ROM (°)	Week 0	103.13 ± 3.72	106.68 ± 2.99
	Week 12	109.17 ± 2.79 **	108.44 ± 2.78
Passive Knee Flexion ROM (°)	Week 0	110.48 ± 3.34	112.32 ± 3.09
	Week 12	114.70 ± 2.61 *	113.08 ± 2.68
Active Knee Extension ROM (°)	Week 0	69.29 ± 2.88	71.22 ± 1.63
	Week 12	78.47 ± 1.31 **	74.70 ± 1.49
Passive Knee Extension ROM (°)	Week 0	73.59 ± 2.44	75.96 ± 1.73
	Week 12	83.12 ± 1.42 **	79.00 ± 1.56

Data are expressed as mean ± SE. Asterisks indicate within-group changes from Week 0 to Week 12 based on paired tests after BH-FDR correction: * *p* < 0.05, ** *p* < 0.01. Between-group comparisons were performed on change scores (Δ Week 12 − Week 0) and are reported in Supplementary Table S3.

**Table 3:** Knee injury and osteoarthritis outcome score (KOOS) subscales—Symptoms, Pain, Function in Daily Living, Function in Sport and Recreation, and Quality of Life.

KOOS	Time	AMS	Placebo
Symptoms	Week 0	59.42 ± 2.73	63.05 ± 2.49
	Week 12	67.42 ± 2.39 **	68.52 ± 2.58
Pain	Week 0	67.95 ± 2.61	72.43 ± 2.51
	Week 12	76.32 ± 3.23 **	74.29 ± 2.36
Function in Daily Living	Week 0	73.47 ± 4.07	75.24 ± 2.51
	Week 12	79.63 ± 3.52 *	75.67 ± 2.45
Function in Sport and Recreation	Week 0	54.47 ± 4.68	61.19 ± 3.62
	Week 12	66.58 ± 3.97 **	66.43 ± 3.09
Quality of Life	Week 0	48.42 ± 3.43	58.19 ± 3.91
	Week 12	57.32 ± 3.35 **	55.38 ± 4.07
Total	Week 0	60.68 ± 2.91	65.90 ± 2.62
	Week 12	69.37 ± 2.92 **	68.00 ± 2.40

Data are expressed as mean ± SE. Asterisks indicate within-group changes from Week 0 to Week 12 based on paired tests after BH-FDR correction: * *p* < 0.05, ** *p* < 0.01. Between-group comparisons were performed on change scores (Δ Week 12 − Week 0) and are reported in Supplementary Table S3.

**Table 4:** Changes in inflammatory cytokine levels.

	Time	AMS	Placebo
IL-8 (pg/mL)	Week 0	3.10 ± 0.53	2.66 ± 0.24
	Week 12	2.32 ± 0.36 *	2.46 ± 0.46
IL-1β (pg/mL)	Week 0	878.62 ± 108.25	924.64 ± 107.273
	Week 12	727.91 ± 125.96	968.29 ± 155.34
MIP-1α (pg/mL)	Week 0	6.34 ± 1.82	5.01 ± 2.77
	Week 12	6.12 ± 1.32	5.15 ± 2.38

Data are expressed as mean ± SE. Asterisks indicate within-group changes from Week 0 to Week 12 based on paired tests after BH-FDR correction: * *p* < 0.05. Between-group comparisons were performed on change scores (Δ Week 12 − Week 0) and are reported in Supplementary Table S3.

**Table 5:** Changes in cartilage degradation and synthesis biomarkers.

	Time	AMS	Placebo
CTXII (ng/mL)	Week 0	2.19 ± 0.55	2.83 ± 0.55
	Week 12	1.10 ± 0.23	2.41 ± 0.58
COMP (ng/mL)	Week 0	4.56 ± 0.76	3.61 ± 0.60
	Week 12	2.38 ± 0.32 *	3.45 ± 0.58
MMP13 (pg/mL)	Week 0	2708.85 ± 299.98	2408.87 ± 318.90
	Week 12	2025.44 ± 234.90 *	2111.50 ± 329.24
COL2A1 (ng/mL)	Week 0	7.66 ± 1.98	9.75 ± 1.74
	Week 12	5.88 ± 1.41	7.90 ± 1.46
PIINP (ng/mL)	Week 0	2.20 ± 0.30	1.80 ± 0.26
	Week 12	2.98 ± 0.33 **	2.01 ± 0.26

Data are expressed as mean ± SE. Asterisks indicate within-group changes from Week 0 to Week 12 based on paired tests after BH-FDR correction: * *p* < 0.05, ** *p* < 0.01. Between-group comparisons were performed on change scores (Δ Week 12 − Week 0) and are reported in Supplementary Table S3.

## Data Availability

The original contributions presented in this study are included in the article. Further inquiries can be directed to the corresponding author.

## Supplementary Materials

The following supporting information can be downloaded at: https://www.mdpi.com/article/10.3390/nu18121842/s1, Table S1: Baseline characteristics and between-group balance check; Table S2: ELISA assay characteristics for serum biomarkers; Table S3: Comprehensive analysis: within-group, between-group, and FDR-corrected; Figure S1: Serum biomarker distributions at baseline and Week 12; Figure S2: HPTLC fingerprint analysis of the AMS test material.
