# Supplementary material for: Evaluation of the Effect of Astragalus membranaceus Saponins Administration on Knee Function and Cartilage Biomarkers in Healthy Subjects with Knee Discomfort
Source: Nutrients. 2026 Jun 7;18(12):1842. doi: 10.3390/nu18121842 (PMC13304541; doi:10.3390/nu18121842)
Supplement: Supplementary file 1 [file nutrients-18-01842-s001.zip › nutrients-4319061-supplementary.pdf]

Supplementary Materials

*Evaluation of the Effect of **Astragalus membranaceus** Saponins Administration on Knee Function and Cartilage Biomarkers in Healthy Subjects with Knee Discomfort*

Supplementary Table S1

Baseline characteristics and between-group balance check

Characteristic	AMS group (n = 23)	Placebo group (n = 25)	p-value
**Demographics**			
Age (yr), mean ± SD	43.3 ± 19.8	50.0 ± 15.2	0.199 (t-test)
Age range (yr)	20 – 67	20 – 70	0.535 (MW-U)
20–39 yr, n (%)	11 (48%)	6 (24%)	
40–49 yr, n (%)	0 (0%)	3 (12%)	
50–59 yr, n (%)	3 (13%)	8 (32%)	
60–75 yr, n (%)	9 (39%)	8 (32%)	
Sex (Female), n (%)	14 (61%)	19 (76%)	0.413 (χ^2)
Sex (Male), n (%)	9 (39%)	6 (24%)	
BMI (kg/m²), mean ± SD	23.6 ± 3.1	24.7 ± 3.4	0.247 (t-test)
**Lifestyle**			
IPAQ activity — Moderate, n (%)	18 (78%)	20 (80%)	1.000 (χ^2)
IPAQ activity — High, n (%)	5 (22%)	5 (20%)	
**Clinical**			
Baseline VAS pain (0–10), mean ± SD	5.5 ± 0.9	5.3 ± 0.6	0.376 (t-test)
**Concomitant therapies during trial**			
NSAID / analgesic use, n (%)	0 (0%)	0 (0%)	—
Other joint supplement use, n (%)	0 (0%)	0 (0%)	—
Major dietary change reported, n (%)	0 (0%)	0 (0%)	—

*Continuous variables compared by two-sample t-test (parametric); Mann–Whitney U test additionally applied to age due to bimodal distribution in the AMS group. Categorical variables compared by chi-square test**. No statistically significant baseline imbalance was observed for any demographic or lifestyle variable (all p > 0.05)**.*

The 6.7-year mean age difference did not reach significance (t-test p = 0.199; MW-U p = 0.118). Throughout the 12-week intervention, no participant in either group reported NSAID/analgesic use, other joint-related supplement use, or major dietary change at any follow-up visit (Weeks 0 and 12); compliance with concomitant-therapy restrictions was therefore complete in both arms.

Supplementary Table S2

ELISA assay characteristics for serum biomarkers

Biomarker	LoD	Intra-CV (%)	Inter-CV (%)	Mean (all samples)	Min observed	Within 2× LoD	Detection rate (%)
IL-8 (pg/mL)	0.10	5.2	8.4	2.63	0.26	0	100.0
IL-1β (pg/mL)	1.00	6.1	10.2	874.9	184.7	0	100.0
MIP-1α (pg/mL)	0.50	7.0	11.1	5.66	0.10	17	92.4
CTX-II (ng/mL)	0.10	4.8	7.9	2.14	0.21	0	100.0
COMP (ng/mL)	0.05	5.5	9.0	3.50	0.65	0	100.0
MMP-13 (pg/mL)	50.0	6.3	10.5	2313.7	368.2	0	100.0
COL2A1 (ng/mL)	0.10	5.9	9.4	7.80	0.41	0	100.0
PIINP (ng/mL)	0.05	5.6	8.7	2.25	0.47	0	100.0

All assays performed in duplicate using commercially available ELISA kits, with quality controls run on each plate. LoD = lower limit of detection (manufacturer's specification). Intra-CV / Inter-CV = within-assay and between-assay coefficients of variation, computed from duplicate measurements and inter-plate quality controls. Mean and minimum values are computed across all measurements. MIP-1α showed values within 2× LoD in 17 samples, reflecting the typically low circulating concentrations of this chemokine; the analytical signal was nonetheless above background for all samples (detection rate 92.4%).

Supplementary Table S3

Comprehensive analysis: within-group, between-group, and FDR-corrected

Part A. Functional and patient-reported outcomes

Endpoint	AMS Δ (p)	AMS q-BH	Placebo Δ (p)	Placebo q-BH	Btw Δ	Btw p	Btw q-BH	Cohen's d
SLSD steps	+12.78 (0.014 *)	0.017 †	+4.48 (0.085 ns)	0.225 ns	+8.30	0.221 ns	0.241 ns	+0.36
Recovery time	-108.91 (0.000 ***)	0.002 †	-56.48 (0.247 ns)	0.412 ns	-52.43	0.220 ns	0.241 ns	-0.36
Active flexion	+6.04 (0.006 **)	0.008 †	+1.76 (0.369 ns)	0.443 ns	+4.28	0.130 ns	0.195 ns	+0.45
Passive flexion	+4.22 (0.027 *)	0.027 †	+0.76 (0.693 ns)	0.756 ns	+3.46	0.191 ns	0.241 ns	+0.38
Active extension	+9.18 (0.001 **)	0.004 †	+3.48 (0.083 ns)	0.225 ns	+6.26	0.051 ns	0.107 ns	+0.66
Passive extension	+9.53 (0.001 ***)	0.002 †	+3.04 (0.113 ns)	0.225 ns	+6.49	0.026 *	0.078 ns	+0.74
KOOS Symptoms	+8.00 (0.002 **)	0.004 †	+5.48 (0.071 ns)	0.225 ns	+2.52	0.489 ns	0.489 ns	+0.22
KOOS Pain	+8.37 (0.001 ***)	0.002 †	+1.86 (0.309 ns)	0.412 ns	+6.51	0.020 *	0.078 ns	+0.77
KOOS ADL	+6.16 (0.019 *)	0.020 †	+0.43 (0.788 ns)	0.788 ns	+5.73	0.053 ns	0.107 ns	+0.65
KOOS Sport/Rec	+12.11 (0.003 **)	0.005 †	+5.24 (0.053 ns)	0.225 ns	+6.87	0.121 ns	0.195 ns	+0.51
KOOS QoL	+8.89 (0.004 **)	0.005 †	-2.81 (0.291 ns)	0.412 ns	+11.70	0.003 **	0.038 †	+1.00
KOOS Total	+8.68 (0.000 ***)	0.002 †	+2.10 (0.110 ns)	0.225 ns	+6.59	0.010 **	0.057 ns	+0.89

Part B. Biomarker outcomes (with FDR correction)

Biomarker	AMS Δ (p)	AMS q-BH	Placebo Δ (p)	Btw p	Btw q-BH	Cohen's d
IL-8	-0.77 (0.003 **)	0.008 †	-0.20 (0.110 ns)	0.049 *	0.158	-0.60
IL-1β	-150.71 (0.344 ns)	0.393	+43.65 (0.791 ns)	0.393 ns	0.593	-0.25
MIP-1α	-0.22 (0.846 ns)	0.846	+0.13 (0.065 ns)	0.751 ns	0.859	-0.09
CTX-II	-1.10 (0.048 *)	0.078	-0.42 (0.566 ns)	0.445 ns	0.593	-0.23
COMP	-2.18 (0.022 *)	0.045 †	-0.16 (0.716 ns)	0.049 *	0.158	-0.60
MMP-13	-683.41 (0.003 **)	0.008 †	-297.36 (0.086 ns)	0.171 ns	0.341	-0.42
COL2A1	-1.77 (0.079 ns)	0.106	-1.84 (0.091 ns)	0.957 ns	0.957	+0.02
PIINP	+0.78 (0.001 ***)	0.008 †	+0.21 (0.054 ns)	0.059 ns	0.158	+0.58

*Within-group changes (Δ AMS, Δ Placebo): mean change from Week 0 to Week 12; paired-sample t-test (if Shapiro–Wilk p > 0.05 on differences) or Wilcoxon signed-rank test otherwise. Between-group analysis: Student's t-test on change scores. Cohen's d: standardized between-group effect size using pooled SD of change scores. Benjamini–Hochberg FDR-adjusted q-values within the eight-biomarker panel. * p < 0.05; ** p < 0.01; *** p < 0.001; † q < 0.05 after FDR correction; ns = not significant.*

Supplementary Figure S1

Serum biomarker distributions at baseline and Week 12

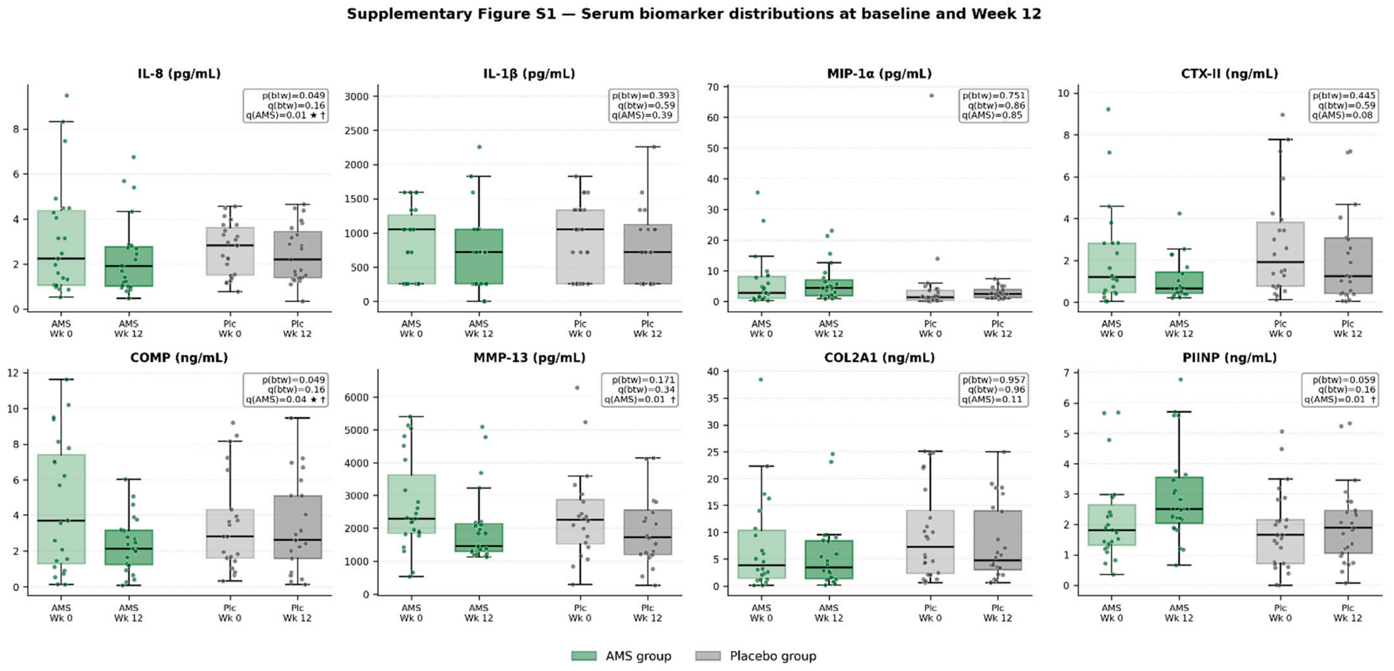

p(btw) = between-group difference in change scores (Welch t-test, uncorrected); q(btw) = Benjamini–Hochberg FDR-adjusted q-value for between-group comparison; q(AMS) = BH-FDR-adjusted q-value for AMS within-group change.
★ between-group p < 0.05; † AMS within-group q < 0.05 after FDR correction

Supplementary Figure S2

Supplementary Figure S2 — HPTLC fingerprint of the AMS test material co-migrating with the astragaloside IV reference standard

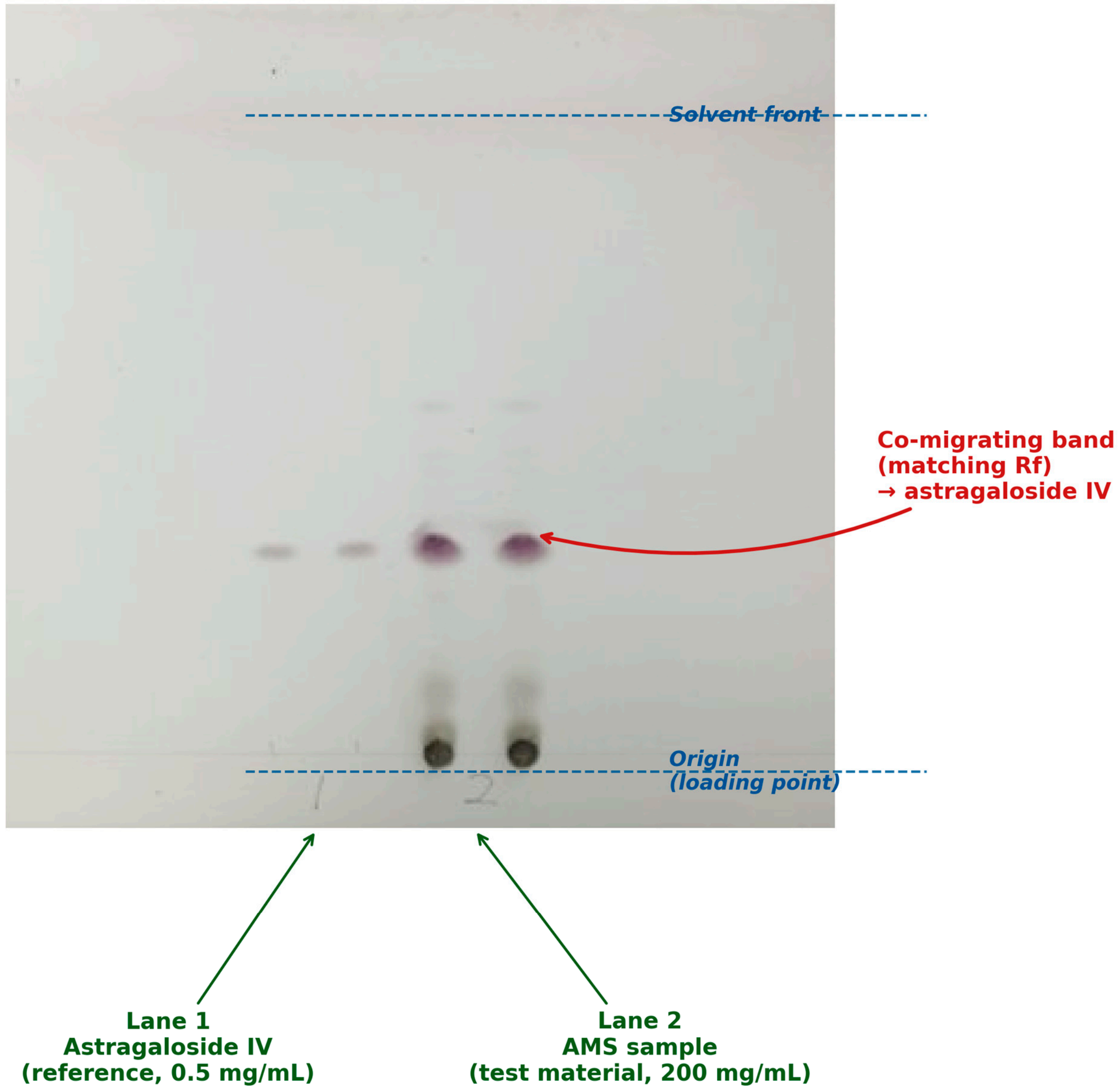

Supplementary Figure S2. HPTLC fingerprint analysis of the AMS test material. Lane 1: astragaloside IV reference standard (0.5 mg/mL in methanol). Lane 2: AMS test material (200 mg/mL in methanol).
